# Assessment of the Portable C-320 Electronic Nose for Discrimination of Nine Insectivorous Bat Species: Implications for Monitoring White-Nose Syndrome

**DOI:** 10.3390/bios10020012

**Published:** 2020-02-13

**Authors:** Anna C. Doty, A. Dan Wilson, Lisa B. Forse, Thomas S. Risch

**Affiliations:** 1 Department of Biology, California State University Bakersfield, Bakersfield, CA 93311, USA; 2 Department of Biological Sciences, Arkansas State University, Jonesboro, AR 72467, USA; trisch@astate.edu; 3 Pathology Department, Southern Hardwoods Laboratory, Southern Research Station, USDA Forest Service, Stoneville, MS 38776, USA; dan.wilson2@usda.gov (A.D.W.); lbforse@gmail.com (L.B.F.)

**Keywords:** chiroptera, White-Nose Syndrome, carbon black polymer composites, electronic aroma detection, noninvasive early disease detection, smellprint signatures, volatile organic compounds (VOCs)

## Abstract

The development of new C-320 electronic-nose (e-nose) methods for pre-symptomatic detection of White-Nose Syndrome (WNS) in bats has required efficacy studies of instrument capabilities to discriminate between major sources of volatile organic compounds (VOCs) derived from clinical samples. In this phase-2 study, we further tested this e-nose for capabilities to distinguish between bat species based on differences in whole-body VOC emissions. Live healthy individuals of nine bat species were temporarily captured outside of caves in Arkansas and Louisiana. VOC emissions from bats were collected using newly developed portable air collection and sampling-chamber devices in tandem. Sensor-array output responses to bat VOC emissions were compared to those of 22 pure VOC analytical standards from five chemical classes. Distinct smellprint signatures were produced from e-nose analyses of VOC metabolites derived from individual bat species. Smellprint patterns were analyzed using 2-dimensional and 3-dimensional Principal Component Analysis (PCA) to produce aroma map plots showing effective discrimination between bat species with high statistical significance. These results demonstrate potential instrument efficacy for distinguishing between species-specific, bat-derived VOC metabolite emissions as major components of clinical samples collected from bats in caves for disease detection prior to symptom development. This study provided additional information required to fully test the efficacy of a portable e-nose instrument for diagnostic applications in subsequent phase-3 testing of noninvasive, early WNS disease detection in intra-cave hibernating bats.

## 1. Introduction

A relatively new disease of Nearctic cave-dwelling bats species, known as White-Nose Syndrome (WNS), first appeared in North American bat populations within caves in New York State in 2006 [1]. This infectious disease is responsible for the reduction of up to 75–99% of bat populations in many winter hibernacula within the eastern half of the continent over the past thirteen years [2,3]. White-Nose Syndrome is caused by cutaneous infections by a nonnative, dermatophytic fungal pathogen, *Pseudogymoascus destructans* (Pd), that induces necrotic skin lesions and spreads primarily through direct and indirect contact between bats and by anthropogenic means [4,5]. Skin infections by Pd disrupt physiological patterns of hibernating bats, causing chronic respiratory acidosis, dehydration, and frequent torpor interruptions that deplete essential winter fat reserves, leading to starvation and other fatal effects [6,7,8,9].

Conservation of insectivorous North American bat species affected by WNS has implications for both agriculture and human health. These small, night-feeding bats consume many costly crop- and tree-insect pests that annually cause millions of dollars in economic losses in the United States and Canada. Insectivorous bats serve as a natural source of biological control agents for the low-cost maintenance of agricultural insect pests, help disrupt the reproductive cycles of damaging insect pests, lower pest-insect population levels below economic thresholds, and reduce the need for toxic insecticides [10,11,12]. Insectivorous bats also help to effectively reduce populations of blood-sucking insects (e.g., mosquitos and biting flies) that vector microbial pathogens responsible for many important human diseases. A wide diversity of mosquito species, consumed by small insectivorous bats, are responsible for the transmission of zoonotic viral pathogens, such as West Nile Virus (WNV) and other arboviruses, carrying them to amplifying bird hosts, other animal reservoirs, and to humans [13,14]. Zinn and Humphrey [15] calculated that a single colony of 30,000 southeastern bats (*Myotis austroriparius*) could consume up to 15 tons of mosquitoes annually, while Rydell [16] estimated that individual insectivorous bats are capable of consuming up to 1000 mosquitoes per hour.

Detecting the presence of diseased bats in early stages of Pd infection is difficult and usually requires tactile and nontactile (primarily audible) semi-invasive disturbance, leading to further depletion of bat fat reserves during hibernation [17,18]. Current methods used to detect the presence of the Pd pathogen (not WNS disease) in caves largely depend on the visual appearance of diseased or symptomatic bats coupled with bat mortality within the cave. Visual cues prompt the use of quantitative polymerase chain reaction (qPCR) DNA amplification, to confirm the presence of Pd mycelium on external bat surfaces [19,20]. After initial infection, WNS mortality does not begin to occur until 2.5 to 3 months later in susceptible species [21]. Although disease prevalence increases sharply from autumn to early winter, peak Pd fungal loads usually do not occur until late winter after symptoms appear [22]. Based on this scenario, WNS-control treatments would not be applied (following qPCR Pd detection) until after disease progress had already reached exponential growth and after secondary Pd-spread had occurred in the cave population. Preliminary results suggest that Pd causes the release of pathogen-specific and disease-associated volatile organic compound (VOC) metabolites into the air surrounding bats at early stages of skin infections before WNS symptoms develop [23,24]. These VOC mixtures potentially are detectable noninvasively with trained electronic nose (e-nose) instruments using specialized reference-library databases [25,26,27,28,29,30,31].

Electronic-nose devices, gas-sensing instruments that utilize sensor arrays capable of detecting complex mixtures of VOCs in air samples by aroma signature patterns (e-nose smellprints), have been used for the detection of microbial pathogens and human diseases in the biomedical field since the mid-1980s [32]. Since then, utilization of e-nose applications for disease detection, referred to as electronic aroma detection (EAD) technologies, have been extended to plant [33,34,35,36,37,38,39,40], animal [41,42,43,44,45,46], and human disease diagnostics [47,48,49,50], forensic pathology [51], clinical disease screenings [26,31], and monitoring human health, medical wastes, and hazardous environmental pollutants [52,53]. Applications of EAD methods for detection of infectious diseases take advantage of the unique metabolic pathways of pathogenic agents that alter the normal physiology of their hosts to produce a wide range of abnormal VOC metabolites not produced by healthy individuals [26,34]. E-nose instruments detect these unique VOC emissions (gaseous mixtures) produced by microbial pathogens and associated diseases, allowing for their early detection and identification.

The science of diagnosing a disease, such as WNS using an e-nose device, requires an accounting of the specific sources of different VOC components present in complex air samples collected from healthy and infected (diseased) individuals. A list of major sources of VOCs associated with noninvasive early WNS disease diagnoses include: (1) normal bat host-derived VOCs collected in air samples released in the exhaled breath and from body surfaces of healthy bats (the sample type collected in this study), (2) volatiles released from hyphae of the Pd pathogen growing on the skin of infected bats, (3) WNS disease induced VOCs produced by alterations in the physiology of Pd-infected bat hosts, and (4) cave-specific background volatiles derived from both dead organic matter and living organisms that release VOCs into cave air. Previous studies (phase-1 research) have already shown that Pd releases specific and detectable VOCs (both primary and secondary metabolites) in vitro from culture headspace that are different from those released by close genetic fungal relatives [23,24]. Fortunately, the probability of achieving effective portable e-nose discriminations of bat species is very high (>95% success rate), given the high-resolving (discrimination) power of the C-320 e-nose tested here and prior demonstrated success of this instrument for numerous biomedical applications [32,47,49,51,54,55,56].

The relatively low cost ($9000–$11,500 base unit price), light weight (0.7 kg or 1.5 lbs.), and portable Cyranose 320 (C-320) e-nose (Sensigent Intelligent Sensing Solutions, Baldwin Park, CA, USA) has been used successfully to detect and identify microbial pathogens responsible for human eye infections at a diagnostic accuracy of 96–98% using Linear Principal Component Analysis (LPCA) and pattern-recognition algorithms [54,55]. The C-320 e-nose also was used to diagnose asthma, a human respiratory (lung) disease, based on abnormal VOC mixtures released in expired human breath [56]. Many other microbial diseases of mammals potentially may be diagnosed through e-nose breath analysis [29,31,49,50]. In this study, we have initiated field testing of the C-320 e-nose for noninvasive early detection of WNS disease in susceptible bat species.

The purpose of the current study (phase-2 research) was to develop a bat VOC signature library, containing reference databases and smellprint signatures specific to the C-320 e-nose, which discriminate between active healthy, insectivorous bat species occurring outside of caves during non-hibernation periods in the Mid-South and Southern regions of the United States. Species-specific smellprint signatures that define healthy active bats will be used in a subsequent study (phase-3 research) for comparisons with intra-cave hibernating healthy and WNS-diseased bats (in various torpor states) of the same species. Our specific objectives were to: (1) develop effective methods for collecting and analyzing air samples from healthy, active bats as well as background extra-cave air (as controls), using the portable C-320 e-nose, (2) establish VOC smellprint signature patterns for nine bat species within defined e-nose-specific databases, and (3) determine the potential capabilities of the C-320 portable e-nose for discriminating between bat species based on statistical analyses of bat VOC smellprint signatures. We hypothesized that differences in the chemical composition of VOC emissions from different bat species would result in distinct e-nose smellprint signatures, and that e-nose sensor responses are differentially determined by exposure to VOCs from different chemical classes. This hypothesis was further tested by exposing the C-320 sensor array to pure VOC standards (from known chemical classes) to assess individual sensor responses for comparisons to VOCs in bat emissions. We surmised that if differences in smellprint signatures were found between bat species, this discrimination capability could be used to facilitate development of new noninvasive methods (not requiring bat handling or captures) for distinguishing between bat species during winter hibernation in caves and for determining the WNS disease status of bats.

## 2. Materials and Methods

### 2.1. Field Sampling Locations and Species

This study was conducted within three parishes in the state of Louisiana and five counties in the state of Arkansas. The majority of bat samples were collected from June–November 2017 (99 samples) and only one sample was collected in April 2018 (from a *Myotis leibii*). Bats were captured in forested landscapes using mist nets (Avinet, Portland, ME, USA) and harp traps (Bat Conservation and Management, Carlisle, PA, USA) set at dusk, or by using a hand net (American Educational Products LLC, Chippewa Falls, WI, USA) at bridges or culverts during daylight hours. We collected 100 air samples from nine species of bats of known sex including: *Corynorhinus rafinesquii*, (7M, 3F, 1 no data); *Eptesicus fuscus* (4M, 18F); *Lasiurus borealis* (9M, 9F); *Lasiurus seminolus* (1M, 2F); *Myotis austroripiarus* (10M, 9F); *Myotis leibii* (1M); *Nycticeius humeralis* (10M); *Perimyotis subflavus* (3M, 2F); and *Tadarida brasiliensis* (4M, 6F) as summarized in Table 1.

All VOC air samples were collected from active bats outside of caves, not during the winter hibernation season. Thus, all bats were nonsymptomatic for WNS and considered to be WNS disease free for the purposes of this study. The Pd pathogen is not known to survive well for extended periods outside of the cave environment following spring bat emergence when Pd incidence and Pd inoculum loads on bats continue to drop dramatically during summer months based on PCR data [58].

### 2.2. Ethical Considerations

All bat sample collection activities were conducted in accordance with the Institutional Animal Care and Use Committee (IACUC) regulations at Arkansas State University (document IACUC # FY16-17-22), using research permit approvals for bat captures from Arkansas Game and Fish Commission (Permit # 051020161) and from Louisiana Department of Wildlife and Fisheries (Permit # LNHP-17-024).

### 2.3. Bat Air Sample Collection Procedures

A 950 mL bat air-sampling chamber was constructed from an inert clear type III soda-lime, tall-profile glass jar with a PTFE-lined polypropylene 89 mm screw-top lid (Manufacturer no. 1211000 Thermo-Fisher Scientific, Waltham, MA). Holes were drilled in the lid and liner to accept two PTFE two-way valves with on/off closures and sealed Viton O-rings with a large-to-small universal connector (Catalog No. OF001101, Kinesis^Tm^ Omnifit^Tm^, Altrincham, Cheshire, UK). The connector with valve accepted 4.8 mm ID, 6.4 mm OD FEP Tubing (Catalog No. TFI-0316-030; Jensen Inert Products, Coral Springs, FL, USA).

An external tube was used to connect the air-sampling chamber (through Port 1) to a 1 L multi-layer foil (pre-filled) pure-air source bag used for pure-air refills and purges. The pure-air bag was comprised of polyethylene and aluminum foil (PE-AL) and contained a screw cap combo valve with septum and 4.8 mm OD valve stem (Item # GSAP001-0707S, Jensen Inert Products, Coral Springs, FL, USA). The pure-air source bag was prefilled with zero-grade ultrapure air (AirGas, a division of Air Liquide, Radnor, PA, USA) containing <0.1 PPM total hydrocarbons. The other end of the tubing extended 152 mm down to the bottom of the air-sampling chamber. Tubing on port 2 (for sample acquisition output) extended ~10 mm into the glass sampling chamber, and was connected on the outside to a Low Vac 1 L vacuum box air sampler (Model 1060, Xitech Instruments, Inc., Placitas, NM, USA), containing an identical PE-AL VOC air sample bag. Alternatively, an Escort Model LC Personal vacuum pump (Zefon International Co., Ocala, FL, USA) was used in combination with a Pelican-case Small Gas Sampling Bag Chamber (Item # ZA0321, Zefon International Co., Ocala, FL, USA).

**Figure 1:**
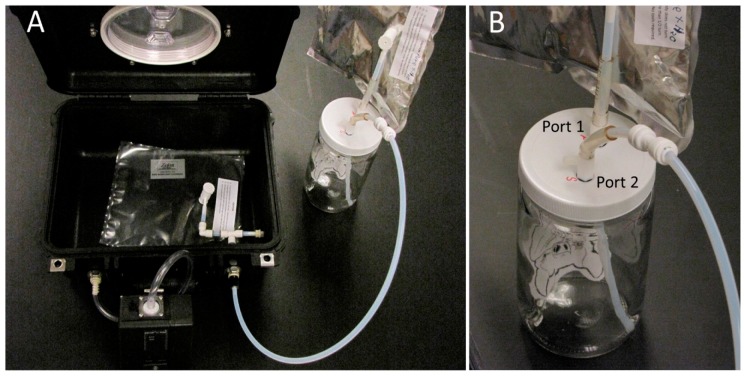
Bat VOC air collection apparatus assembly. (**A**) Pelican case vacuum chamber (containing PE-AL VOC air-sampling bag) with bat air-sampling chamber and Escort vacuum pump attached to vacuum chamber ports (left photo), (**B**) Close-up of bat air-sampling chamber with Port 1 (connected to pure zero-air replacement bag), and Port 2 (connected to outflow sample air tube attached to input port on Pelican case vacuum chamber (right photo).

The Xitech vacuum box air sample design allowed the VOC air sample to move directly from the source bat air sampling chamber into the VOC air sample bag (inside the vacuum box) without coming into direct contact with the pump, maintaining VOC sample integrity and avoiding VOC cross-contamination between samples. The complete assembled bat air-sampling chamber, used in combination with the Escort vacuum pump and Pelican case vacuum box chamber, is illustrated in Figure 1.

Individual bats were placed into the sampling chamber immediately after capture and held for ~10 min with port valves set in the “off” (closed) position, to create headspace and isolate the bats from ambient air for precise controlled sampling. The sampling chamber was covered with a cloth bag during headspace buildup to minimize stress to bats. Following bat-VOC headspace buildup, both valves were switched “on” (open to air flow) and the vacuum pump to the vacuum-pump box was turned on to create a vacuum within the vacuum box, causing the VOC air sample bag to inflate due to air being pulled out of the bat sampling chamber at a rate of 0.5 L/min from port 2 (sample acquisition port) into a fresh VOC air sample bag. Ultra-zero pure air connected to port 1 (pure-air refill port) replaced air removed from the sampling chamber at the same rate to ensure outside air did not contaminate the sample while equalizing pressure within the sampling chamber containing the live bat.

Following sampling, zero-grade ultrapure air was pulled through the sampling tubing using the vacuum box to purge the lines and sampling chamber of any VOCs before and/or after each sample acquisition. Individual sampling chambers were used for a single bat for each sampling night. Sampling chambers were washed and/or autoclaved for re-use in subsequent sampling events. All air samples in VOC collection bags were transported overnight to the USDA Forest Service, Southern Hardwoods Laboratory (SHL, Stoneville, MS, USA) pathology laboratory for e-nose analyses.

Bats removed from the sampling chamber were subsequently processed and measured to acquire information of sex, reproductive status, age, mass (g), and forearm length (cm). The entire procedure from bat capture to release took <20 min per sampled individual. Bats from Arkansas were immediately released following VOC headspace extraction and processing. Bats from Louisiana were euthanized and collected as part of an agreement between personnel from Texas Tech University and the Louisiana Department of Wildlife and Fisheries.

### 2.4. C-320 E-Nose Sensor Array and Preliminary VOC Testing

The portable C-320 e-nose (Cyranose 320, Sensigent, Baldwin Park, CA, USA), tested for efficacy in discriminating nine separate bat species in this study, contained a 32-sensor array consisting of carbon black polymer composite (CBPC) sensors coated with different types of cross-reactive proprietary conducting polymers (CPs). The typical types of C-320 sensor-array outputs and associated smellprint signatures from this unit were initially compared using a 90 s Manual-test mode run in response to zero-grade ultra-pure moist air (UPMA) sample and 60 s sample runs with known VOC pure chemical standards run in the training mode.

The relative sensitivity of individual thirty-two numbered sensors (S1–S32) within the C-320 sensor array preliminarily was tested using purified chemical standards (Sigma-Aldrich Corp., St. Louis, MO, USA) from each of five chemical classes: including alcohols, aldehydes, amines, carboxylic acids, and ketones, compared with UPMA control, to assess probable correlated responses to VOC analytes present in air samples collected from individual bat species. The following twenty-two total chemical standards were tested from the following chemical classes: (6 alcohols) methanol, ethanol, 1-propanol, 2-propanol, 1-butanol, and t-butanol; (6 aldehydes) formaldehyde, acetaldehyde, propionaldehyde, crotonaldehyde, furfuraldehyde, and benzaldehyde; (2 amines), cyclohexylamine and diethanolamine; (4 carboxylic acids) formic acid, acetic acid, lactic acid, and butanoic acid; and (4 ketones) acetone, butanone, 2-octanone, and 3-octanone. In each case, 50 μL of a chemical standard was placed into a 1 L clear bag prefilled with zero-grade ultrapure air (UPA) without moisture. Sample bags were heated in a Model 750F oven (Fisher Scientific, Pittsburg, PA, USA) at 35 °C for 90 min prior to C-320 e-nose analysis.

### 2.5. Bat Air Sample Preparation for E-Nose Analysis

Field air sample bags collected from each bat species were injected with 0.50 mL of autoclaved, double-distilled and multi-filtered 18 mΩ ultrapure water (Millipore Model Milli-Q UV-Plus, Molsheim, France) and heated in a Model 750F oven (Fisher Scientific, Pittsburg, PA, USA) at 35 °C for 90 min, then cooled to 21 °C immediately prior to e-nose analysis. The addition of moisture to each sample provided a stable, consistent continuity to baseline sensor responses of the C-320 sensor array, preconditioned with UPMA by Manual test-mode runs prior to air sample analyses, to assure that sensor responses were not significantly affected by differences in moisture content of field bat air samples.

### 2.6. Bat VOC Sample E-Nose Analysis

Initial analysis of VOCs in bat air samples using the C-320 electronic nose was conducted in the training mode. A minimum of five sample runs were required for determination of smellprint signatures for VOC metabolites of each bat species, but in most cases smellprint signatures were obtained from at least ten samples per bat species (sample type). E-nose data from individual bat species were pooled across all sampling locations because low intra-species variability resulted in distinct data clusters by species in PCA plots. Test groups analyzed in the C-320 training mode allowed a maximum of six sample types (bat species) for calculations of PCA of species tested within each test group. Consequently, in order to accommodate all nine sample types (bat species) in addition to control ambient air samples, multiple test groups were created with sample types placed in different combinations for each test group created. The result was a separate PCA analyses for each test group.

The three functional ports of the portable C-320 e-nose instrument consisted of: (1) the chamber purge port (right side), (2) the exit port (left side) and (3) the sample introduction or input port (top-right side of instrument). The exit port was left open for exhaustion of air from the sensor array during analyses unless individual samples were to be recovered for subsequent chemical analyses in which case a new VOC collection bag was attached to the exit port during sample input only. An UPMA bag was attached to the chamber-purge port. A luer lock Stopcock 316 stainless steel 3-way valve [FLL, MLL, FLL (left) orientation] tubing connector (Item # 6017SS, Cadence Science Corp., Cranston, RI, USA) was attached to the C-320 top input port to allow for introduction of sample air from the VOC sample bags during sample intake into the instrument and for air purges through the input port after sample acquisitions; and to completely close the valve between sample runs. The sensor array was purged with UPMA using Manual test mode between runs of different sample types to prevent carryover cross-contamination of VOCs between different bat species during analyses. A secondary UPMA purge bag was attached to the secondary port on the 3-way luer lock valve to allow for pure-air purges of the input port following sample introductions. A 25 mm 0.45 μm PTFE syringe filter (Item # 09-719H, Fisher Scientific, Pittsburg, PA, USA) was placed between the pure-air purge bags and the purge port as well as between the VOC sample bag and the sample entry port, using short segments of green 4.0 mm ID, 6.0 mm OD TFE tubing (Item # TMT0406-040, Jensen Inert Products, Coral Springs, FL, USA), to prevent entry of small particulates into the instrument during analyses.

The C-320 run cycle established for all bat air sample analyses included the following sequential steps: an initial 5 s UMPA input port baseline purge at low pump speed, followed by a 30 s sample intake (draw #1) run at high pump speed during which sample analyte VOCs adsorbed to the surfaces of individual sensors in the sensor array providing sensor outputs, then 10 s each of UPMA purges again at low pump speed from the input port and then the sample intake ports, respectively; with a total run time per cycle of approximately 55 s. Sensors S5 and S31 on the sensor array were turned off for bat air samples because these sensors usually produced very large responses that were disproportionate to the other 30 sensors in the array.

The method run parameters used for all analytical runs, specified for training sets consisting of six sample types each, utilized only one training repeat count and one identifying repeat count setting. The substrate heater was turned on and set at 35 °C and digital filtering was always turned on for training, identifying, and calibration modes. Data recording settings selected for all analytical methods were as follows: canonical algorithm, auto-scaling preprocessing of data, data normalization #1 method, medium identification quality (for sample discriminations), and 99.9% acceptance threshold. Symbols for sensor polarities and normalized highest mean sensor response intensity (msri) ranges of individual sensors within smellprint signatures for the C-320 sensor array are described in Table 2. Sensor response intensities indicate the mean change in electrical resistance recorded by each sensor (in response to all adsorbed VOCs), relative to the base-resistance condition when no VOC are adsorbed to the CP-coating on the CBPC sensor surface. The msri values of all sensors are assembled together and represented by *y*-axis values for all 32 individual sensors in the C320 sensor array as quantified (using bar length) in the data output bar graph (which collectively represent the smellprint pattern).

### 2.7. Statistical Analysis of E-Nose Data

The production of output smellprint signatures from the sensor array for each bat species was done using plotting methods that provided mean responses (black bars) of the highest digital value of sensor responses for all replications that occurred during analysis runs with sensor response range variations shown by gray area with hash marks, indicating ±1 standard error of the mean (±SEM). Additionally, sensor S6 was not displayed in most sensor run-output graphs, but was included in data analyses, because of the very large intensity response that obscured the displayed intensity responses of the other sensors, but not to the same intensity as observed in Sensors S5 and S31.

Each test group was done using different combinations of six sample types per test group, and analyzed separately using single-class 3-dimensional Principal Component Analysis (PCA) and secondary statistics including 2-dimensional or 3-dimensional Canonical Discriminant Analysis Cross Validation (CDA-CV), and by SIMCA Cross Validation (SIMCA-CV). Statistical analyses of all e-nose data (as metafiles) were performed using Chemometric Data Analysis Program (CDAnalysis) vers. 9.5 software (Sensigent, Baldwin Park, CA, USA). Aroma map axes, defined as Euclidean distances, represented principal components of aroma signatures and provided the percentages of the total variance represented by each principal component. Statistical levels of discrimination measured between sample types within each test group, based on differences in VOC metabolite composition of air samples from each bat species, were determined using PCA and CDA-CV primary statistics, followed by secondary analyses of class ownership (bat VOC profiles) for each sample class using SIMCA-CV and Hierarchical Cluster Analysis (HCA) with K-mean nearest-neighbor group method to produce dendrogram plots of aroma class groupings.

## 3. Results

### 3.1. Sensory Outputs of the C-320 E-Nose Sensor Array

The C-320 e-nose, like most electronic-nose devices, contains a sensor array with multiple sensors that each respond differentially, depending on sensor-coating type, to all VOC analytes present in the air sample being analyzed. Each sensor in the 32-sensor array is cross-reactive with different levels of sensitivity to specific VOCs depending on their chemical class, relative abundance (concentration), and relative composition (molar ratio) within the sample. Individual sensor responses to different sample types are determined by the net response to all VOC types and quantities present.

**Figure 2:**
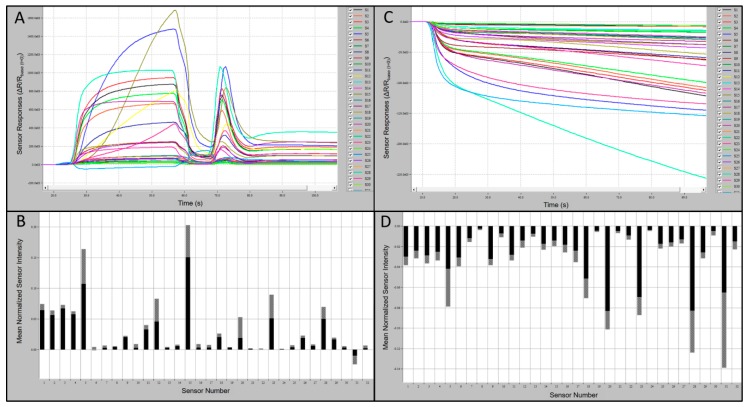
Portable C-320 e-nose output runs and associated smellprint signatures. (**A**) Analysis run output from 60 s sample runs in the training test mode for 1-butanol VOC (alcohol chemical class); (**B**) Smellprint signature of 1-butanol from the 32-sensor array, normalized mean highest sensor responses in black (±SEM, gray error bar) during runs of 10 replicate samples; (**C**) Sensor output responses to a zero-grade ultra-pure moist air (UPMA) sample during a 90 s manual-test mode run; (**D**) Smellprint signature of UPMA from the 32-sensor array ran in the analytical training test mode, normalized mean highest sensor responses in black (±SEM, gray error bar) during runs of 10 replicate samples.

Preliminary tests of the C-320 e-nose sensor array provided indications of relative-intensity responses of individual sensors to known VOCs compared to UPMA used as a control. Typical sensor-array outputs and associated smellprint signatures from the C-320 e-nose in response to chemical standards, using 1-butanol (alcohol chemical class) as an example, are presented in Figure 2A-D. Sensor responses from analysis of 1-butanol were strongly positive, up to 1000 × 10^3^ highest mean sensor response intensity (msri) for most sensors and 1200–1650 × 10^3^ for sensors S5 and S15, with the only exception of sensor S31 which was mildly negative (Figure 2A). The associated aroma smellprint pattern of 1-butanol contained equivalent normalized msri equivalent values ranging from <0.10 for most sensors to up to 0.20 for sensors S5 and S15 (Figure 2B). By contrast, sensor-array output responses to UPMA were consistently negative in the absence of all VOCs with relatively mild negative responses <−150 msri for most sensors and up to −250 for sensor S31 (Figure 2C). The corresponding smellprint signature for UPMA similarly was mildly negative with mean normalized sensor responses of <−0.14 for all sensors (Figure 2D).

C-320 sensor array analysis output responses to VOCs had varying polarity of responses to individual chemical standards that were not consistent in smellprint signatures for all test compounds within each chemical class. Some compounds within a chemical class produced largely negative responses of all sensors in the array, whereas other compounds in the same chemical class produced mostly positive-polarity responses for most sensors.

Sensor output responses of certain individual sensors, particularly sensors S5, S6, and S31, during analyses of VOCs from different chemical classes vs. bat air sample types yielded different results. Sensor responses of all three sensors to VOCs generally occurred within the normal range of responses, including msri ranges recorded for VOCs, whereas responses of these sensors to bat VOCs were quite different. Sensors S5 and S31 provided overwhelming responses to bat VOCs that largely overpowered and obscured the other sensors in the array precluding effective discriminations. Similarly, sensor S6 usually produced a strong negative response to complex mixtures of VOC metabolites from most bat species.

### 3.2. C-320 E-Nose Preliminary VOC Testing

The C-320 e-nose sensor array produced smellprint patterns of sensor responses that varied considerably in both polarity and intensity to VOCs from different chemical classes. Sensor responses of most sensors in the C-320 e-nose sensor array generally provided mostly positive responses to representative VOCs derived from the five chemical classes tested (alcohols, aldehydes, amines, carboxylic acids, and ketones). However, responses of the sensor array to specific compounds from each chemical class also often produced variable-polarity responses depending on the chemical nature of individual compounds tested (Table 2). Sensors S6, S22, and S31 consistently produced negative responses to alcohols and aldehydes, whereas sensors S5 and S17 yielded negative responses to only aldehydes, and sensor S17 produced consistently negative output responses to amines and ketones. However, most sensors in the C-320 array gave positive responses to ketones with the exceptions of nine sensors including S5–7, S9, S16–17, S22–23, and S26. The sensor array produced consistently negative output responses for all sensors to UPMA control samples which contained no VOCs within highly purified air. The presence of moisture (water vapor) within pure air samples did not significantly affect sensor responses compared with UPA alone.

Smellprint normalized sensor intensity responses to VOCs from different chemical classes, unlike polarity (+, ±, or −) responses, varied more frequently between individual sensors in the C-320 array rather than between chemical classes (Table 2). Very low (VL) mean sensor response intensity (msri) ranges generally were produced in all five chemical classes for sensors S7–S10, whereas low (LW) msri responses to all five chemical classes were recorded for sensors S1–4. Moderate (MD) msri-range responses to alcohols, aldehydes, and carboxylic acids for sensors S5, S15, S23, S28 and S31; and in response to ketones for sensors S11–12, and S28. A high (HI) msri-range response was recorded only for alcohols for sensor S6. Very high (VH) msri-range responses were produced for carboxylic acids and ketones for sensor S31. There were no extremely high (EH) intensity range (0.300–0.399 msri) or ultrahigh (UH)-level (≥0.400 msri) range responses for any VOCs from among the five chemical classes tested.

Sensor response intensity ranges of the C-320 sensor array to UPMA control samples yielded normalized msri-range responses within smellprint signatures that were negative for all sensors unlike sensor-array responses to known chemical-standard VOCs from all chemical classes tested (Table 2). The vast majority of sensor responses of individual sensors were recorded in the very low (VL) intensity range (0.001–0.049 msri) category. The unique combinations of intensity and polarity responses of individual sensors within smellprint signatures provided detailed criteria to facilitate discriminations of chemical analytes according to chemical classes. This information was subsequently used in the analysis of bat air samples collected from VOC emissions for obtaining qualitative information about the possible chemical identities (by chemical class) of VOC composition of gaseous constituents in sampled air.

### 3.3. Field Bat C-320 E-Nose Outputs and Smellprint Signatures

**Figure 3:**
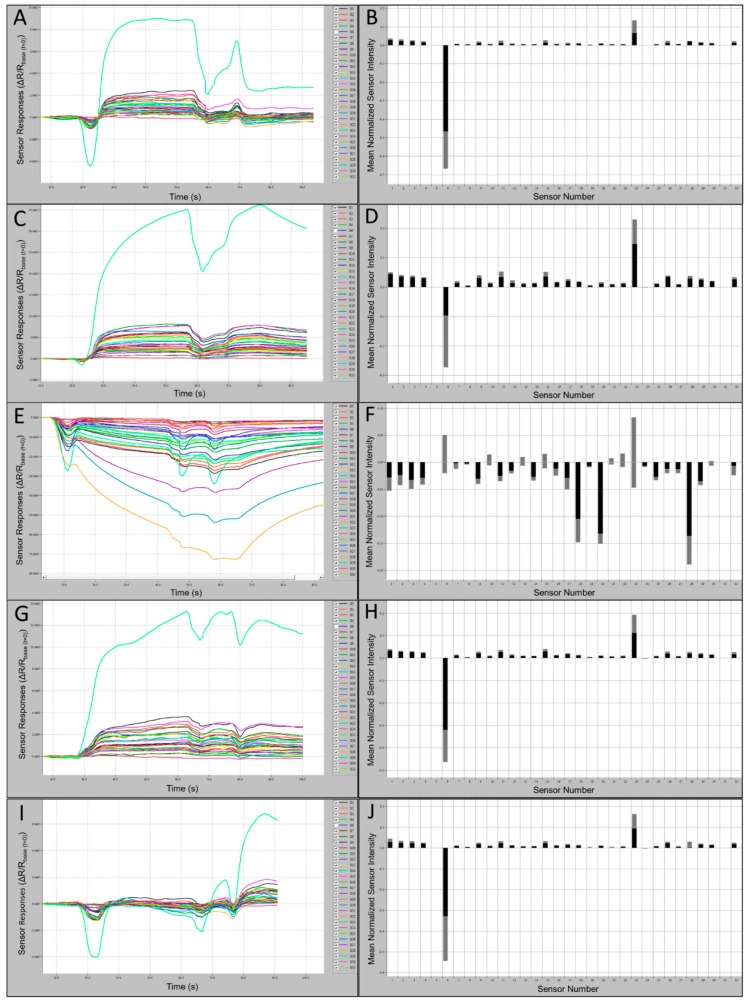
Bat air sample 60 s e-nose analysis runs with 32-sensor outputs and associated smellprint signatures. (**A**) Sensor-array outputs for *Eptesicus fuscus* (EPFU); (**B**) Smellprint signature of *Eptesicus fuscus*; (**C**) Sensor outputs for *Myotis austroripiarus* (MYAU); (**D**) Smellprint signature of *Myotis austroripiarus*; (**E**) Sensor outputs for *Myotis leibii* (MYLE); (**F**) Smellprint signature of *Myotis leibii*; (**G**) Sensor outputs for *Perimyotis subflavus* (PESU); (**H**) Smellprint signature of *Perimyotis subflavus*; (**I**) Sensor outputs for ambient-air control (CTRL) samples from field bat collection sites; (**J**) Smellprint signature of CTRL air samples. Sensor S6 is not displayed due to overly intense responses that obscure all other sensors. Smellprint bar graphs indicate mean sensor responses in black (±SEM, gray error bar).

Comparisons of C320 e-nose output runs and associated smellprint signatures, generated from nine field bat species, provided evidence of differences in total VOC metabolite emissions from individual species. Subtle differences in sensor intensities and polarities across the sensor array provided cumulative variations yielding distinctive smellprint patterns for each species. Sensor S6 produced consistently negative responses and sensor S23 produced consistently positive responses to VOCs of all species tested. Sensory data outputs recorded during analytical runs along with corresponding smellprint signatures are presented in Figure 3. Sensor responses were viewed on the sensor response curves as different colors.

Samples from all species, with the exception of MYLE, caused positive responses from all or most sensors. The greatest positive sustained response from a single sensor over the range of species and the control was to Sensor 23, and the most negative response was from Sensor 6 with the exception of MYLE VOCs, which caused the most negative response from Sensor 28 and a positive response from Sensor 6. Samples from all species, with the exception of MYLE, demonstrated a normalized msri > 0.1 from Sensor 23, while all other sensors responses were <0.1. Sensor-array output intensities (oi ≤ 2.2 × 10^3^) and smellprint (normalized msri < 0.03) for most sensor responses to EPFU VOCs were attenuated due to relatively low concentrations of total VOC emissions (Figure 3A,B). By contrast, most sensor responses to MYAUvolatiles were significantly stronger with output intensities (up to 7.5 × 10^3^) and smellprint msri < 0.05 (Figure 3C,D). Additionally, MYAU samples demonstrated a second highest normalized msri from Sensor 11 (green in Figure 3C), followed by Sensor 15 then Sensor 1 (Figure 3C,D). These small differences in responses to sensors and mean intensity may indicate strong compositional differences in VOC emissions between species. LABO presented the highest positive mean sensor response to Sensor 23, being >0.3 (Figure 3D), while MYLE VOCs presented the lowest positive mean sensor intensity response from Sensor 23, being <0.1 (Figure 3F). The majority of sensor responses to MYLE VOCs were strongly negative (oi ≤ −72.0 × 10^3^) with a strikingly different smellprint signature with msri ≤ −0.18 (Figure 3E,F). Sensor outputs from VOCs of PESU were moderate in intensities (oi ≤ 3.9 × 10^3^) and smellprint (msri < 0.04) (Figure 3G,H). Sensor array responses to ambient-air control (CTRL) samples from field bat collection sites yielded weak output signals (oi ≤ 2.0 × 10^3^) and a reduced smellprint, indicated by msri < 0.03 (Figure 3I,J).

Sample gas-vapor relative humidity (RH) had relatively minor effects on C-320 sensor array responses compared with all VOCs tested from bats and analytical standard samples. However, sensor intensities did increase proportional to VOC concentrations in air samples analyzed. Also, VOC gas analytes appeared to separate by density of individual VOC components present within sample bags, causing greater variability of sensor responses between replicate runs, if sample bags were not heated to increase uniformity of VOC concentrations and RH within sample bags.

**Figure 4:**
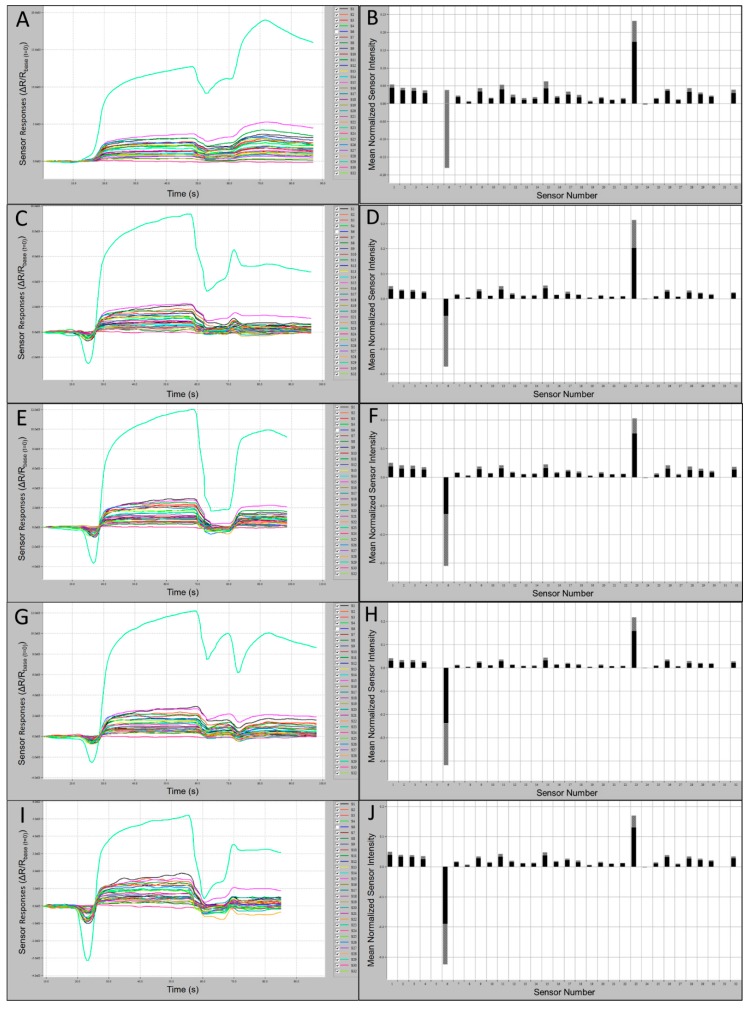
Bat air sample 60 s e-nose analysis runs with 32-sensor outputs and associated smellprint signatures. (**A**) Sensor-array outputs for *Corynorhinus rafinesquii* (CORA); (**B**) Smellprint signature of *Corynorhinus rafinesquii*; (**C**) Sensor outputs for *Lasiurus borealis* (LABO); (**D**) Smellprint signature of *Lasiurus borealis*; (**E**) Sensor outputs for *Lasiurus seminolus* (LASE); (**F**) Smellprint signature of *Lasiurus seminolus*; (**G**) Sensor outputs for *Nycticeius humeralis* (NYHU); (**H**) Smellprint signature of *Nycticeius humeralis*; (**I**) Sensor outputs for *Tadarida brasiliensis* (TABR); (**J**) Smellprint signature of *Tadarida brasiliensis*. Sensor S6 is not displayed. Smellprint bar graphs indicate mean sensor responses in black (±SEM, gray error bar).

Sensor output intensities for VOCs of CORA were strongly positive (oi ≤ 4.0 × 10^3^) and smellprint (msri < 0.06) for most sensor responses (Figure 4A,B). Weaker sensor intensities were recorded for volatiles from LABO, including sensor outputs of oi ≤ 2.1 × 10^3^ and smellprint (msri < 0.05) for most sensor responses (Figure 4C,D). Subtle differences between sensor magnitude responses from individual species also indicated interspecific distinctions in VOC composition and intensity. For example, for species LABO, Sensor 15 occupied the second-highest normalized msri, followed by Sensor 1. Slightly greater output intensities (oi ≤ 3.0 × 10^3^) and smellprint (msri < 0.06) were measured for VOCs of LASE displayed in (Figure 4E,F). Similar but slightly weaker sensor responses (oi ≤ 2.3 × 10^3^) and smellprint (msri < 0.03) were observed in response to volatiles of NYHU indicated in Figure 4G,H. However, sensor responses to TABR VOCs showed reduced output intensities (oi ≤ 1.9 × 10^3^) and smellprint (msri < 0.04) shown in Figure 4I,J.

### 3.4. Discriminations of Field Bat Species VOC Emissions 3.4.1 Test Group 1 Analyses

**Figure 5:**
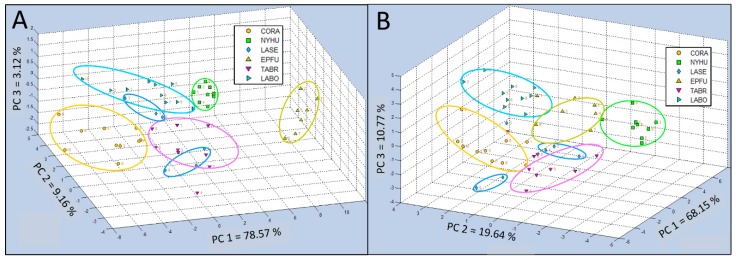
Comparison of C-320 e-nose VOC profiles of bat air samples from six species (Test Group 1) using 3-dimensional Principal Component Analysis (PCA) and Canonical Discriminant Analysis Cross Validation (CDA-CV). (**A**) Single-class PCA of VOC emissions from *Corynorhinus rafinesquii* (CORA), *Nycticeius humeralis* (NYHU), *Lasiurus seminolus* (LASE), *Eptesicus fuscus* (EPFU), *Tadarida brasiliensis* (TABR), and *Lasiurus borealis* (LABO); (**B**) CDA-CV of VOC emissions from the same six bat species in Test Group 1.

The spatial distribution of data clusters for six bat air sample classes derived from 3-dimensional PCA tests, displayed as an aroma map score plot for bat air VOCs in Test Group 1 (including CORA, NYHU, LASE, EPFU, TABR, and LABO species), showed that all of the aroma classes were separated into distinct clusters, despite cases of apparent false class overlaps that were resolved using plot rotations to clarify spatial class differentiations (Figure 5A). In all cases of PCA tests of e-nose smellprint data, principal components (PCs) of chemical constituents, present in complex VOC bat air samples of each chemical class, were represented in PCA data plots as follows: PC 1 = *z*-axis, PC 2 = *x*-axis, and PC 3 = *y*-axis. Percent values for each PC indicate the percentage of total variance, accounting for the variability explained by each principal component (PC), as follows: PC 1 = 78.57%, PC 2 = 9.16%, and PC 3 = 3.12%. Scaled units indicated for all axes were measured as Euclidean distance for PCA and Canonical distance for CDA-CV.

The 3-dimensional single-class PCA of Test Group 1 showed bat VOC emissions of the EPFU class were least chemically related to the other five classes. VOCs of the CORA class were at the other end of the spectrum furthest away from the EPFU class, but more closely located to the remaining four classes (NYHU, LASE, TABR, and LABO). The replicate data points for the LASE class were split into two distinct groups.

The results of CDA-CV for Test Group 1 generally provided indications of less tightly clustered groupings and more widely dispersed data points within classes, improving class definitions (Figure 5B). However, the LASE class remained fractured and was not effectively cross validated but consolidated into a coherent cluster by canonical analysis. Distribution of variances among three PCs were more evenly distributed with PC 1 = 68.15%, PC 2 = 19.64%, and PC 3 = 10.77%, indicating significant complexity in bat VOC emission components requiring more principal components to explain differences in aroma class chemical composition.

**Figure 6:**
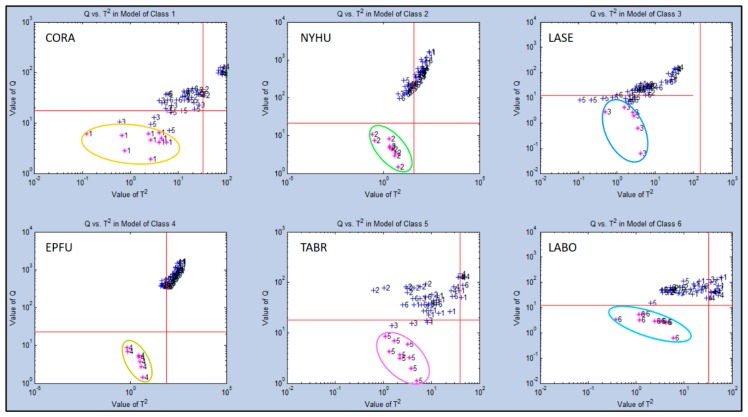
Analysis of aroma class ownership of C-320 e-nose VOC profiles based on bat air samples from six species (Test Group 1) using SIMCA Cross Validation (SIMCA-CV) secondary statistical tests. Analysis of Q-distance (Q-value) vs. score distance (T^2^-value) for each individual VOC aroma class (compared to all other classes) defined by VOC emissions from Class 1 = *Corynorhinus rafinesquii* (CORA), Class 2 = *Nycticeius humeralis* (NYHU), Class 3 = *Lasiurus seminolus* (LASE), Class 4 = *Eptesicus fuscus* (EPFU), Class 5 = *Tadarida brasiliensis* (TABR), and Class 6 = *Lasiurus borealis* (LABO).

Further secondary statistical evaluation of Test Group 1 using SIMCA-CV provided means for evaluating individual single classes. This was done by comparisons with the other five classes to indicate precise quantitative differences in class ownership and aroma chemical-relatedness distances between individual classes using Q-distance (Q-values) vs. score distance (T^2^-values) to generate aroma discrimination distances between data points for each aroma class (Figure 6). The greatest discrimination distance occurred for the EPFU aroma class (Q = 10^3^) which was most separated from the other five aroma classes, confirming results with PCA data. The next most distant aroma class was the NYHU class (Q = 10^2^–10^3^). Intermediate levels of discrimination distances were found for CORA, TABR, and LABO at near (Q = 10^1^–10^2^). The smallest discrimination distance (Q = 10^1^) was found for the LASE aroma class, suggesting a data cluster found near the center of all other aroma classes in PCA plots, but still significantly distinct.

#### Test Group 2 Analyses

**Figure 7:**
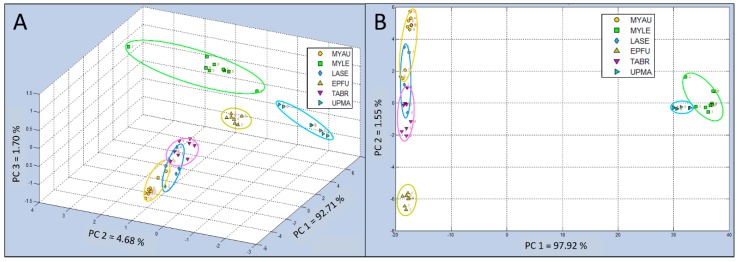
Comparison of C-320 e-nose VOC profiles of bat air samples from (Test Group 2) using 3-dimensional Principal Component Analysis (PCA) and 2-dimensional Canonical Discriminant Analysis Cross Validation (CDA-CV). (**A**) Single-class PCA of VOC emissions from *Myotis austroripiarus* (MYAU), *Myotis leibii* (MYLE), *Lasiurus seminolus* (LASE), *Eptesicus fuscus* (EPFU), *Tadarida brasiliensis* (TABR), and Ultrapure Moist Air (UPMA) control; (**B**) CDA-CV of VOC emissions from the same five bat species and control in Test Group 2.

The 3-dimensional single-class PCA of Test Group 2 (including MYAU, MYLE, LASE, EPFU, TABR, and UPMA control sample types), indicated that three of the aroma classes (MYLE, EPFU, and UPMA) were well separated into distinct data clusters, whereas the other three aroma classes (MYAU, LASE, and TABR) had closely-distributed data clusters, indicating more similar chemical relatedness in VOC composition within bat air emissions (Figure 7A). Identified PCs of chemical constituents in VOC bat air samples indicated that the percentage of total variance, accounting for the variability in this test, explained by principal components were as follows: PC 1 = 92.71%, PC 2 = 4.68%, and PC 3 = 1.70%.

Canonical analysis results using 2-dimensional CDA-CV for Test Group 2 generally provided indications of less tightly clustered groupings and more widely dispersed data points within classes, improving class definitions (Figure 7B). The total variance in this analysis was attributed to only two PCs, reducing the analysis to only 2-dimensional, with PC 1 accounting for 97.92% and the remaining very small portion of the variance indicated for PC 2 of only 1.55%. Two aroma classes (MYLE and UPMA) were widely separated from the other four sample classes (MYAU, LASE, EPFU, and TABR).

The EPFU aroma class was well separated from the nearest data clusters (MYAU, LASE, and TABR) which appeared to be somewhat overlapped (both MYAU and TABR with LASE) in 2-dimensional space, but no overlap of class memberships between samples of MYAU and TABR were revealed.

**Figure 8:**
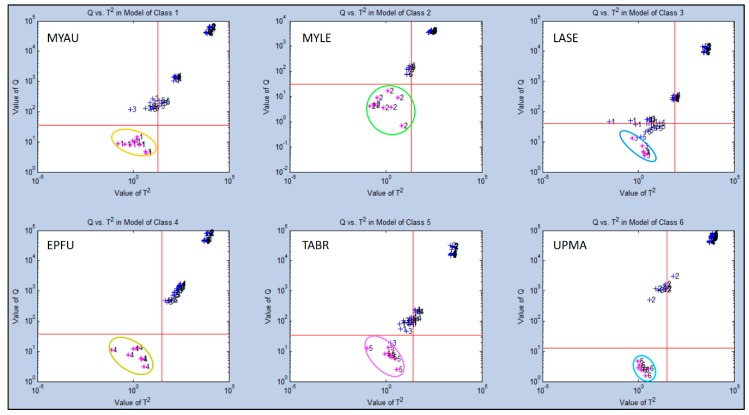
Analysis of aroma class ownership of C-320 e-nose VOC profiles based on bat air samples from five species and ultrapure-air control (Test Group 2) using SIMCA Cross Validation (SIMCA-CV) secondary statistical tests. Analysis of Q-distance (Q-value) vs. score distance (T^2^-value) for each individual VOC aroma class (compared to all other classes) defined by VOC emissions from Class 1 = *Myotis austroripiarus* (MYAU), Class 2 = *Myotis leibii* (MYLE), Class 3 = *Lasiurus seminolus* (LASE), Class 4 = *Eptesicus fuscus* (EPFU), Class 5 = *Tadarida brasiliensis* (TABR), and Class 6 = Ultrapure Moist Air (UPMA) control.

Secondary statistical evaluation using SIMCA-CV for Test Group 2 generated different results from Test Group 1 in quantifying class memberships of individual aroma sample types with all other sample classes. The highest discrimination distances were indicated for UPMA and EPFU (Q = 10^3^–10^5^) both highly separated in class memberships from the other four sample types (Figure 8). The two next most distant aroma classes were MYAU and TABR (Q = 10^2^–10^5^) with strong differences from other classes.

An intermediate level of discrimination distance was found for MYLE (Q = 10^2^–10^4^). The smallest discrimination distance (Q = 10^1^–10^4^) from the other five classes was found for the LASE aroma class, but still distinct even from near-cluster samples of other aroma classes. Again, these results were consistent with PCA and CDA-CV plots showing relative chemical relatedness of VOC composition between aroma classes (sample types).

A third set of six bat samples (Test Group 3) also were tested by C-320 analyses with similar statistical results. The data derived from Test Group 3 is presented in Appendix A. Additional statistical evaluations of distances between centers of numbered aroma-class data clusters on PCA-aroma plots are provided in Supplementary Materials (Figures S1–S3). Supplemental data from statistical analyses of Test Group 4 are provided in Figures S4–S7.

## 4. Discussion

Preliminary investigations into specific sensory responses of the C-320 e-nose sensor array to representative VOCs from five chemical classes provided a basis for obtaining tentative possible identities of unknown VOCs present in bat emissions. The unique response patterns, measured as intensity and polarity of individual sensors within the array to VOCs from different chemical classes, provided criteria for discriminating the probable chemical composition of some VOC sample analytes likely present in bat air samples. Differences in sensor response intensities and polarity across the 32-sensor array provided 64 pieces of data for discriminating and categorizing C-320 response patterns to known individual analytical chemical standards representing each chemical class.

Differential normalized smellprint sensor responses of individual C-320 sensors to specific chemical classes provided important information about the chemical sensitivity of the sensor array potentially useful for determining suitability for specific VOC detection applications. Sensor intensity responses of the C-320 sensor array to bat VOC emissions also suggest that the relative presence of VOCs from specific chemical classes when comparing bat smellprints and sensor response curves to known purified VOCs. The highly positive response of the C-320 array to bat VOCs for sensors which responded positively to the presence of the alcohol chemical class was not surprsing. Bats have a complex makeup of free fatty acids, including fatty alcohols on the skin epidermis [59,60,61], some of which may have antifungal properties with implications for controlling growth of Pd [62]. A number of likely known carboxylic acids, such as lactic acid and carbonic acid, possibly contributed to the detection of VOC emissions that included representatives from this chemical class as a result of bat flight activity. Most bats were captured in flight via mist nets or harp traps. The buildup of carboxylic acids in the bloodstream and released through expelled breath following flight exercise likely accounts for the e-nose detection of carboxylic acids in bat air samples.

Sensors S1–S4 generally responded positively to VOC emission of all bat species, but there were no strong indications of differential responses from these sensors to any of the chemical classes, suggesting limited contributions to bat discriminations based on VOC profiles. Additionally, sensors S5 and S31 were not useful as components within bat smellprint patterns for effective discrimination of bat species in our study, despite having very high normalized responses to alcohols, carboxylic acids, and ketones. Further research is needed to explain the possible reasons why a positive response for Sensor S1 was detected, and minimal response for sensor S31 was indicated in our study, particularly as alcohols, carboxylic acids were both relatively important for all bat species, and ketones for MYAU. This study, to our knowledge, is the first to assess the whole-body VOC composition of bats. Our approach using e-nose technologies allow complex VOC emissions of bats to be recorded as a single, unique species-specific smellprint signature. The potential use of bat VOC emissions for noninvasive species identifications and early disease detections within caves has many advantages over all conventional methods that require the semi-invasive touching or handling of bats which induce unnecessary arousals during hibernation. In addition, specific VOCs identified as disease biomarkers often appear early, prior to symptom development [26,27,49]. Little information was found in the literature concerning research that has explored the VOC emissions of insectivorous bats in relation to disease, behavior, and host physiology. The identification and characterization of unique VOC emissions from individual bat species, recognized here as major components of WNS disease-associated diagnostic e-nose smellprint signatures, is required for developing EAD technologies as a new noninvasive strategy for early diagnosis of the disease. Diagnostic air samples taken from healthy summer field-caught bats within enclosed sampling chambers provided strong baseline data of normal VOC metabolites that may be used for comparisons within VOCs collected in air samples from the close vicinity of healthy and diseased bats in winter hibernacula. Our results from analysis of C-320 e-nose output runs, associated smellprint signatures, and statistical analyses of VOC profiles have provided evidence to demonstrate significant differences in the VOC metabolite emissions from different bat species. These results confirm our operating hypothesis that different bat species smell differently in their healthy state, due to differences in types and amounts of VOC metabolites released from their bodies and breath. These differences in VOC emissions may be used as a basis for discriminating between species based on their unique e-nose smellprint signature patterns. Differences in the chemical composition of VOC profiles determined for individual bat species have been confirmed subsequently by chemical analyses using a dual-technology laboratory-grade GC/E-nose instrument (unpublished data).

Analysis of an air sample collected from the Eastern small-footed myotis (MYLE) was dramatically different from the other bat species studied. We were only able to collect one individual sample of this species. The lack of sufficient replicate samples may indicate that the sensor array output runs and smellprint signatures determined may not be representative of the species. Analysis of additional samples will be required to confirm the VOC profile and smellprint signature of MYLE.

Response of sensor S11 to VOC emissions from the Southeastern myotis (MYAU) were more positive than VOCs of other bat species. This sensor is sensitive to ketones. The production and release of ketones during arousal from torpor and hibernation have been indicated for some small mammals, such as the thirteen-lined ground squirrel (*Spermophilus tridecemlineatus*) and deer mice (*Peromyscus maniculatus*) [63,64]. The majority of MYAU air samples collected in this current study were captured immediately following roost exit on a cold evening in the fall, possibly indicating that ketone levels were elevated in this species due to upregulation to stimulate arousal.

Comparisons of aroma-class smellprint signatures using PCA, CDA-CV, SIMCA-CV and HCA types of analyses provided different tests and ways for demonstrating the discrimination power of this e-nose for these specific sample types. The levels of discrimination determined between bat species and control samples (UPMA, CTRL) were variable for each of the eleven individual aroma classes (nine bat species and two controls). The relative e-nose discrimination potential achieved for individual aroma classes, when compared with all other aroma classes tested with the C-320 e-nose (based on data from four test groups), were found to follow the approximate order (from lowest to highest discrimination) of: LASE < CTRL, CORA, PESU, TABR < LABO < NYHU < MYLE < MYAU < UPMA < EPFU by aroma classes (air sample types). Notice that four aroma classes (CTRL, CORA, PESU, TABR) could be discriminated at approximately the same level from all other sample types, just above LASE which was the aroma class that the C-320 sensor array could least distinguish from the other classes. By comparison, the e-nose had the strongest capability of distinguishing between samples of the EPFU aroma class from all the others tested.

The initial primary steps involved in developing an early, noninvasive WNS disease-detection method based on EAD technologies, require the identification of all sources and specific types of individual VOC components and background VOCs which are present in diagnostic air samples. Discrimination between VOC components from all significant source types should be possible for thorough efficacy testing of e-nose technologies for early disease detection; thus, aptly forming the basis of our research working hypothesis. Initial study results have been favorable in suggesting the strong possibility for achieving a new electronic nose-based diagnostic tool for early WNS disease detection as well as many other recognized potential applications of e-nose technologies for WNS-related studies and disease management [65,66].

The discrimination of bat species by their VOC emissions is critical to chemically defining diagnostic samples for individual species because differences in bat VOC metabolites likely account for the largest variable component among chemical disease biomarkers found in WNS-smellprint signatures. The other significant VOC components in WNS disease-specific smellprint signatures, including VOC metabolites of the Pd pathogen and hibernacula-specific background VOCs, theoretically are less significant because Pd-associated metabolites are probably relatively conserved across geographically-diverse allopatric strains, thought to be the result of a monophyletic introduction [67].

The development of early disease-detection technologies for WNS is essential for achieving more effective means of monitoring and improving disease management. Current technologies for determining WNS disease diagnoses require post-mortem microscopic testing of bat skin and observing diagnostic symptoms [6,8,68,69,70]. The development of a new method for early diagnostic testing for WNS, prior to symptom development, would provide a means of implementing more effective direct control measures to suppress the Pd pathogen, reduce the adverse effects of the disease, and help facilitate the prevention of secondary cycles of bat-to-bat transmission through direct contact and release of Pd-inoculum from infected skin. Also, bats treated prior to symptom development have significantly better chances of recovery from WNS, due to interruption of pathogenesis, when successfully checked by early more effective prophylactic and therapeutic treatments.

## 5. Conclusions

We have determined in this phase-2 study that different healthy bat species produce and release distinct, complex mixtures of normal, healthy-state VOC metabolites which reflect significant compositional differences in VOC profiles. The relative positions and magnitude of C-320 sensor output responses to bat VOC emissions demonstrated distinctly different VOC metabolite components in sampled air, indicated by unique smellprint signatures for each bat species tested. We suspect corresponding smellprint signatures of WNS-diseased bats of each species are significantly different, even at early stages of pathogenesis prior to symptom development, due to WNS-mediated metabolic changes that result in differences in primary disease-associated bat VOCs (alterations in normal host pathways due to pathogenesis and pathogen-associated VOCs). Consequently, differences in VOC profiles determined by e-nose analyses between healthy and diseased individuals potentially establishes a theoretical basis for further testing of e-nose devices as new tools for determining and monitoring the health state of bats, particularly during winter hibernation when they are most vulnerable to damage by WNS.

Previous phase-1 studies have identified principal VOC metabolites produced by Pd in culture [23,24]. Nevertheless, VOC metabolites produced by Pd on bat skins of different bat species, a uniquely different substrate from culture media, must be determined because these may be significantly different from VOCs produce in axenic cultures. However, disease-associated VOCs generated by WNS pathogenesis in different bat species is another significant component that has not yet been studied and accounted for as a major variable component of the WNS disease smellprint signature associated with different bat species. These bat-specific disease biomarker metabolites (abnormal and/or quantitatively different metabolites) in combination with unaltered species-specific metabolites will be the next focus for phase 3 of this research.

## Figures and Tables

**Table 1:** Locations and bat species from which VOC air samples were collected and indications of relative susceptibility to White-Nose Syndrome.

Common Name	Species	Code ^1^	WNS-Relative Susceptibility ^2^	Louisiana			Arkansas					
				Parish			County					
				Grant	Rapides	Natchitoches	Craighead	Logan	Searcy	Washington	Woodruff	Total
Rafinesque’s big-eared bat	*Corynorhinus rafinesquii*	CORA	Pd positive	12								**12**
Big brown bat	*Eptesicus fuscus*	EPFU	Confirmed	1	11				10			**22**
Eastern red bat	*Lasiurus borealis*	LABO	Pd positive				16			2		**18**
Seminole bat	*Lasiurus seminolus*	LASE	Unknown	1		2						**3**
Southeastern myotis	*Myotis austroriparius*	MYAU	Confirmed, pending confirmation of species	4							15	**19**
Eastern small-footed myotis	*Myotis leibii*	MYLE	Confirmed					1				**1**
Evening bat	*Nycticeius humeralis*	NYHU	Unknown				2			8		**10**
Tri-colored bat	*Perimyotis subflavus*	PESU	Confirmed	1		1	2			1		**5**
Mexican free-tailed bat	*Tadarida brasiliensis*	TABR	Pd positive		10							**10**

^1^ Species codes as defined in Table 6.1 of Loeb et al. [57] (p. 38). ^2^ Relative susceptibility rating to WNS: Confirmed = Bat species identified with diagnostic symptoms of WNS; Pd positive = Bat species and subspecies on which *Pd* has been detected, but no diagnostic sign of WNS has been documented (susceptibility ratings from http://whitenosesyndrome.org).

**Table 2:** Normalized mean sensor response intensity ranges within C-320 smellprint signatures of volatile organic compounds from five chemical classes.

	C-320 E-Nose Smellprint Normalized Mean Sensor Responses ^1^																															
Chemical Class ^2^	1	2	3	4	5	6	7	8	9	10	11	12	13	14	15	16	17	18	19	20	21	22	23	24	25	26	27	28	29	30	31	32
Alcohols	**±**	**±**	**±**	**±**	**±**	**−**	**±**	**+**	**±**	**±**	**±**	**±**	**±**	**±**	**±**	**±**	**±**	**±**	**±**	**±**	**±**	**−**	**±**	**±**	**±**	**±**	**±**	**±**	**±**	**±**	**−**	**±**
	**LW**	**LW**	**LW**	**LW**	**MD**	**HI**	**VL**	**VL**	**VL**	**VL**	**VL**	**LW**	**VL**	**VL**	**MD**	**VL**	**VL**	**LW**	**VL**	**VL**	**VL**	**VL**	**MD**	**VL**	**VL**	**VL**	**VL**	**MD**	**VL**	**VL**	**VH**	**VL**
Aldehydes	**±**	**±**	**±**	**±**	**−**	**−**	**±**	**±**	**±**	**±**	**±**	**±**	**±**	**±**	**±**	**±**	**−**	**±**	**±**	**±**	**±**	**−**	**±**	**±**	**±**	**±**	**±**	**±**	**±**	**±**	**−**	**±**
	**LW**	**LW**	**LW**	**LW**	**MD**	**LW**	**VL**	**VL**	**VL**	**VL**	**LW**	**LW**	**VL**	**VL**	**LW**	**VL**	**VL**	**LW**	**VL**	**LW**	**VL**	**VL**	**MD**	**VL**	**VL**	**VL**	**VL**	**LW**	**VL**	**VL**	**MD**	**VL**
Amines	**±**	**±**	**±**	**±**	**±**	**±**	**±**	**±**	**±**	**±**	**±**	**±**	**±**	**±**	**±**	**±**	**±**	**±**	**±**	**±**	**±**	**±**	**±**	**±**	**±**	**±**	**±**	**±**	**±**	**±**	**−**	**±**
	**LW**	**LW**	**LW**	**LW**	**LW**	**VL**	**VL**	**VL**	**LW**	**VL**	**LW**	**LW**	**VL**	**VL**	**LW**	**VL**	**VL**	**LW**	**VL**	**VL**	**VL**	**VL**	**MD**	**VL**	**VL**	**VL**	**VL**	**LW**	**VL**	**VL**	**MD**	**VL**
Carboxylic acids	**±**	**±**	**±**	**±**	**±**	**±**	**±**	**±**	**±**	**±**	**±**	**±**	**±**	**±**	**±**	**±**	**±**	**±**	**±**	**±**	**±**	**±**	**±**	**±**	**±**	**±**	**±**	**±**	**±**	**±**	**±**	**±**
	**LW**	**LW**	**LW**	**VL**	**MD**	**VL**	**VL**	**VL**	**VL**	**VL**	**VL**	**VL**	**VL**	**VL**	**MD**	**VL**	**VL**	**VL**	**VL**	**VL**	**VL**	**VL**	**VH**	**VL**	**VL**	**VL**	**VL**	**MD**	**VL**	**VL**	**VH**	**VL**
Ketones	**+**	**+**	**+**	**+**	**±**	**±**	**±**	**+**	**±**	**+**	**+**	**+**	**+**	**+**	**+**	**±**	**±**	**+**	**+**	**+**	**+**	**±**	**±**	**+**	**+**	**±**	**+**	**+**	**+**	**+**	**−**	**+**
	**LW**	**LW**	**LW**	**LW**	**LW**	**VL**	**VL**	**VL**	**VL**	**VL**	**MD**	**MD**	**VL**	**VL**	**VL**	**VL**	**VL**	**VL**	**VL**	**VL**	**VL**	**VL**	**LW**	**VL**	**VL**	**VL**	**VL**	**MD**	**VL**	**VL**	**VH**	**VL**
UPMA Control	**−**	**−**	**−**	**−**	**−**	**−**	**−**	**−**	**−**	**−**	**−**	**−**	**−**	**−**	**−**	**−**	**−**	**−**	**−**	**−**	**−**	**−**	**−**	**−**	**−**	**−**	**−**	**−**	**−**	**−**	**−**	**−**
	**VL**	**VL**	**VL**	**VL**	**LW**	**VL**	**VL**	**VL**	**VL**	**VL**	**VL**	**VL**	**VL**	**VL**	**VL**	**VL**	**VL**	**LW**	**VL**	**MD**	**VL**	**VL**	**LW**	**VL**	**VL**	**VL**	**VL**	**MD**	**VL**	**VL**	**MD**	**VL**

^1^ Smellprint sensor responses to representative VOCs (from five chemical classes). Symbols for sensor polarities and normalized highest mean sensor response intensity (msri) ranges of individual sensors in the model C-320 e-nose 32-sensor array: **+** = positive sensor response; **–** = negative sensor response; **±** = both positive and negative sensor responses to individual VOCs (within each chemical class). Intensity ranges (with color codes): VL = very low (0.001–0.049 msri, yellow), LW = low (0.050–0.099 msri, cyan), MD = moderate (0.100–0.149 msri, green); HI = high (0.150–0.199 msri, magenta); VH = very high (0.200–0.299 msri, no highlight). ^2^ Individual VOCs (analyzed with the C-320 sensor array) from some chemical classes had exceptionally unusual sensor response (smellprint) patterns that were quite different from responses typical of the majority of VOCs analyzed which were representative of compounds within each individual chemical class; UPMA = ultra-pure moist air.

**Figure A1:**
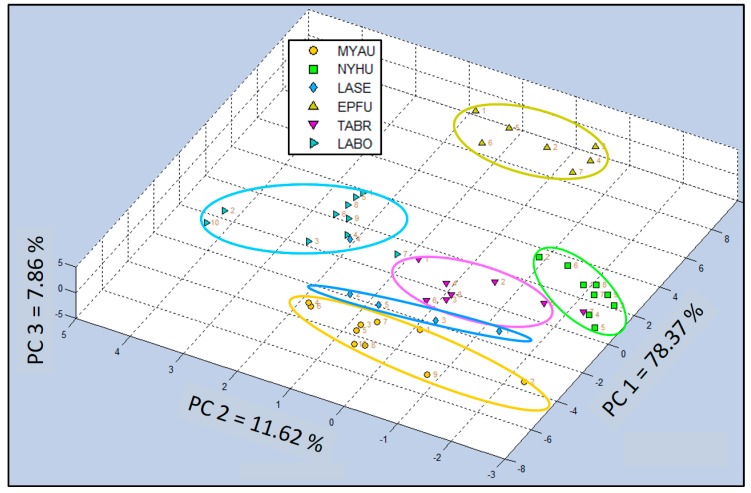
Comparison of C-320 e-nose VOC profiles of bat air samples from six species (Test Group 3) using 3-dimensional Canonical Discriminant Analysis Cross Validation (CDA-CV). Analysis of VOC emissions from *Myotis austroripiarus* (MYAU), *Nycticeius humeralis* (NYHU), *Lasiurus seminolus* (LASE), *Eptesicus fuscus* (EPFU), *Tadarida brasiliensis* (TABR), and *Lasiurus borealis* (LABO).

## Supplementary Materials

The following are available online at https://www.mdpi.com/2079-6374/10/2/12/s1, derived from statistical analyses of C-320 e-nose smellprint data derived from Bat air VOC emissions and controls for the following test groups: Figure S1: HCA (Test Group 1), Figure S2: HCA (Test Group 2), Figure S3: HCA (Test Group 3), Figure S4: HCA (Test Group 4), Figure S5: PCA (Test Group 4), Figure S6: CDA-CV (Test Group 4), and Figure S7: SIMCA-CV (Test Group 4).

## Appendix A

Designations of C-320 E-Nose Method Names—[For Analytical Standards, Pure Chemical Tests]:

Method 1 = **ChemAlco1** for Alcohol Chemical Class; Aroma classes 1–6: (MeOH, EtOH, 1-PrOH, 2-PrOH, 1-BuOH, t-BuOH); Symbol abbreviations: MeOH = methanol; EtOH = ethanol; 1-PrOH = n-propanol, 2-PrOH = 2-propanol, 1-BuOH = n-butanol, t-BuOH = tert.-butanol; Figure 2A, Table 2.
Method 2 = **ChemAldeh2** for Aldehyde Chemical Class; Aroma classes 1–6: (FoHO, AcHO, PrHO, CrHO, FuHO, BeHO); Symbol abbreviations: FoHO = formaldehyde, AcHO = acetaldehyde, PrHO = propionaldehyde, CrHO = crotonaldehyde, FuHO = furfuraldehyde, BeAl = benzaldehyde; Table 2.
Method 3 = **ChemCacid3** for Carboxylic Acids Chemical Class; Aroma classes 1–4: (FoCOOH, AcCOOH, LaCOOH, BuCOOH); Symbol abbreviations: FoCOOH = formic acid, AcCOOH = acetic acid, LaCOOH = lactic acid, BuCOOH = n-butanoic acid; Table 2.
Method 4 = **ChemKeton4** for Ketone Chemical Class; Aroma classes 1–4: (AcCO, BuCO, 2-OcCO, 3-OcCO); Symbol abbreviations: AcCO = acetone, BuCO = butanone, 2-OcCO = 2-octanone, 3-OcCO = 3-octanone; Table 2.
Method 5 = **ChemAmine5** for Amine Chemical Class; Aroma classes 1–2: (CyHN, DeHN); Symbol abbreviations: CyHN = cyclohexylamine, DeHN = diethanolamine; Table 2.
Method 6 = **ChemUPMA6** for Ultrapure Moist Zero-air Control (no VOCs); Aroma class 1: (UPMA); Symbol abbreviation: UPMA = ultrapure moist air; Figure 2B, Table 2.

Designations of C-320 E-Nose Method Names—[For Bat Test Groups 1–3]:

Method 1 = **M5EBatsVC3** for Bat Test Group 1, Aroma classes 1–6: (CORA, NYHU, LASE, EPFU, TABR, LABO); Figure 5, Figure 6, and S1 (Supplementary Materials).
Method 2 = **M7EBatsVC5** for Bat Test Group 2, Aroma classes 1–6: (MYAU, MYLE, LASE, EPFU, TABR, UPMA); Figure 7, Figure 8, and S2 (Supplementary Materials).
Method 3 = **M5EBatsVC4** for Bat Test Group 3, Aroma classes 1–6: (MYAU, NYHU, LASE, EPFU, TABR, LABO); Figures A1 and S3 (Supplementary Materials).
Method 4 = **M5EBatsVC1** for Bat Test Group 4, Aroma classes 1–6: (LABO, NYHU, PESU, EPFU, MYAU, CTRL); Figures S4–S7 (Supplementary Materials).

Bat Test Group 3—Canonical Discriminant Analysis Cross Validation (CDA-CV):

Supplementary data for comparisons of C-320 e-nose VOC profiles of bat air samples from six species (Test Group 3) using 3-dimensional Canonical Discriminant Analysis Cross Validation (CDA-CV). These data are a final replicate of tests, representing experimental results from the third test group (combination of six bat species), which shows a different perspective of the spatial distribution of sample type classes (bat species) indicating chemical relatedness of VOC emissions. Test group 3 CDA-CV data analyses indicates more clearly the position of the VOC emissions signature of the Seminole bat (LASE) to be between the Mexican free-tailed bat (TABR) and the Southeastern myotis (MYAU). Data points for LASE in this analysis showed that all individuals in the LASE class were clustered together similar to Test Group 2, but not split into two separate data clusters as shown in Test Group 1 (Figure A1).
