# Supplementary figures and images for: Assessment of the Portable C-320 Electronic Nose for Discrimination of Nine Insectivorous Bat Species: Implications for Monitoring White-Nose Syndrome
Source: Biosensors (Basel). 2020 Feb 13;10(2):12. doi: 10.3390/bios10020012 (PMC7168176; doi:10.3390/bios10020012)

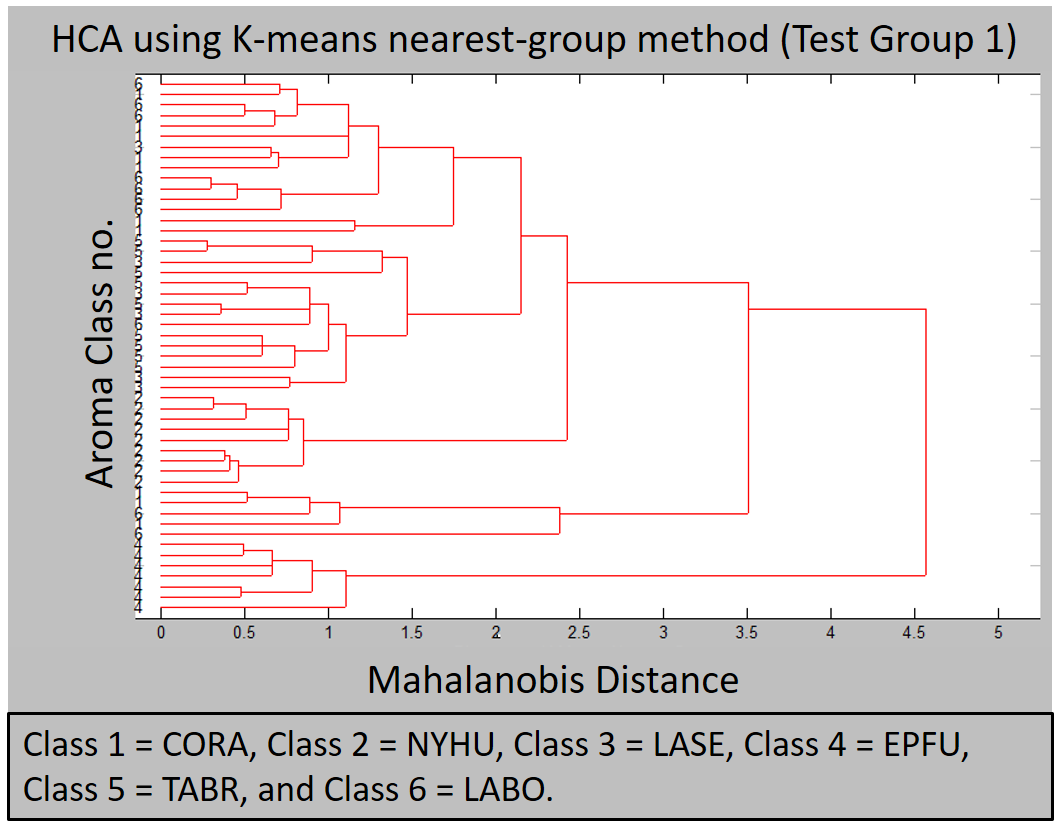

Supplement: Supplementary file 1 [file biosensors-10-00012-s001.zip › Supplementary Figures/Figure S1 - HCA K-mean on Test Group 1.bmp]

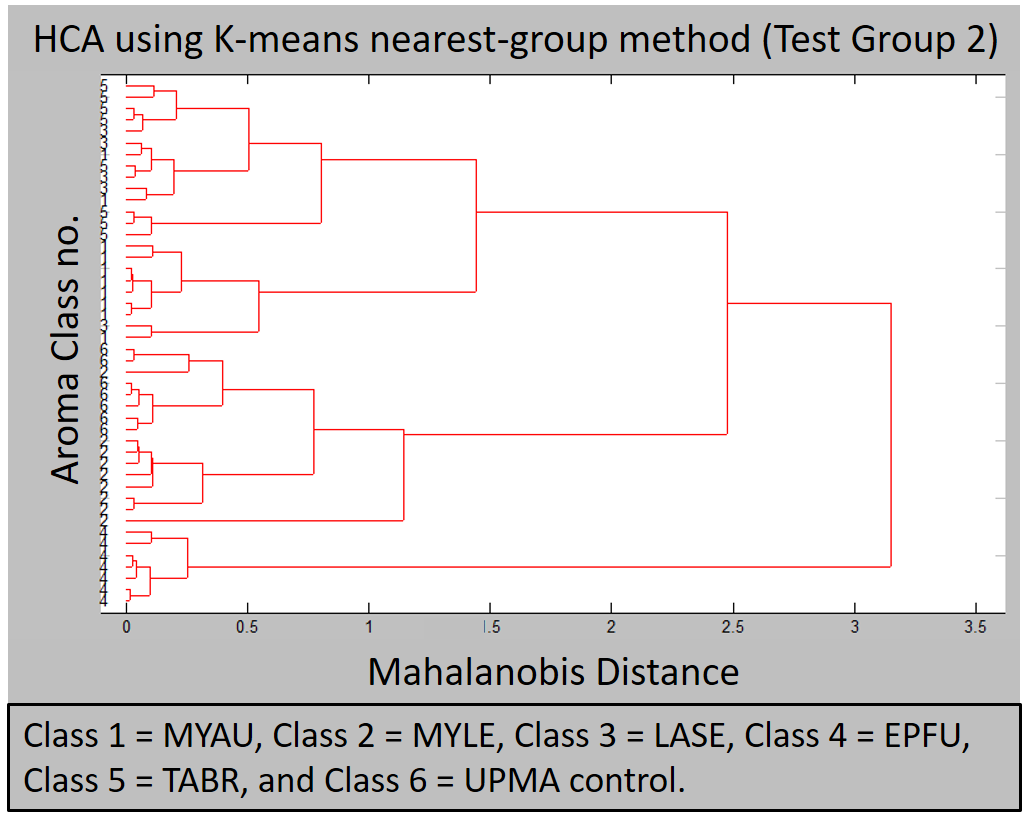

Supplement: Supplementary file 1 [file biosensors-10-00012-s001.zip › Supplementary Figures/Figure S2 - HCA K-mean on Test Group 2.bmp]

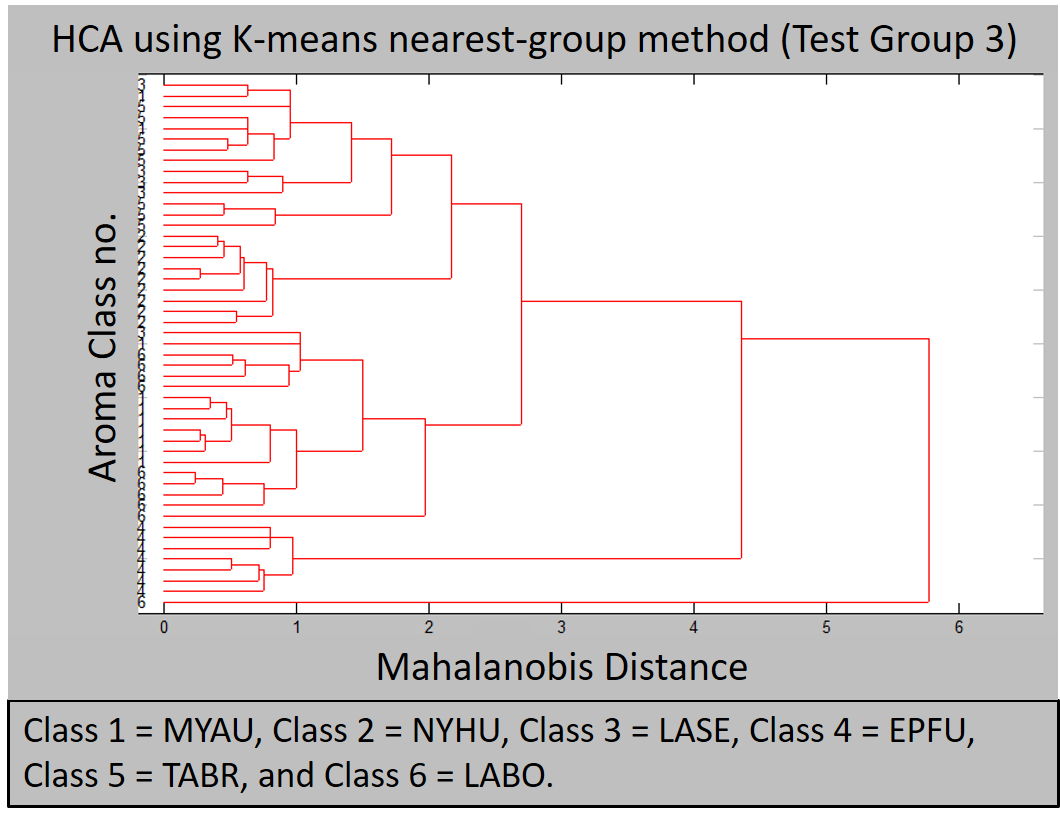

Supplement: Supplementary file 1 [file biosensors-10-00012-s001.zip › Supplementary Figures/Figure S3 - HCA K-mean on Test Group 3.bmp]
